# *Blastocystis* load mediates the gut microbiome associations with within-host diversity of *Blastocystis* in non-human primates

**DOI:** 10.1093/ismeco/ycaf170

**Published:** 2025-09-26

**Authors:** Pingping Ma, Wenjie Mu, Yugui Wang, Yihui Liu, Yang Zou, Zhilong Lu, Shifu Pang, Hong Pan, Long Zhang, Lixian Chen, Yongpeng Yang, Xiaoqi Lin, Zhong Kuang, Weifei Luo, Guohua Liu, Shuai Wang

**Affiliations:** State Key Laboratory of Animal Disease Control and Prevention, College of Veterinary Medicine, Lanzhou University, Lanzhou Veterinary Research Institute, Chinese Academy of Agricultural Sciences, Lanzhou, Gansu 730000, China; State Key Laboratory of Animal Disease Control and Prevention, College of Veterinary Medicine, Lanzhou University, Lanzhou Veterinary Research Institute, Chinese Academy of Agricultural Sciences, Lanzhou, Gansu 730000, China; State Key Laboratory of Animal Disease Control and Prevention, College of Veterinary Medicine, Lanzhou University, Lanzhou Veterinary Research Institute, Chinese Academy of Agricultural Sciences, Lanzhou, Gansu 730000, China; State Key Laboratory of Animal Disease Control and Prevention, College of Veterinary Medicine, Lanzhou University, Lanzhou Veterinary Research Institute, Chinese Academy of Agricultural Sciences, Lanzhou, Gansu 730000, China; State Key Laboratory of Animal Disease Control and Prevention, College of Veterinary Medicine, Lanzhou University, Lanzhou Veterinary Research Institute, Chinese Academy of Agricultural Sciences, Lanzhou, Gansu 730000, China; Guangxi Key Laboratory of Longevity Science and Technology, AIage Life Science Corporation Ltd., Nanning, Guangxi 530200, China; Guangxi Key Laboratory of Longevity Science and Technology, AIage Life Science Corporation Ltd., Nanning, Guangxi 530200, China; Guangxi Key Laboratory of Longevity Science and Technology, AIage Life Science Corporation Ltd., Nanning, Guangxi 530200, China; Kunming Biomed International, Kunming, Yunnan 650500, China; Kunming Biomed International, Kunming, Yunnan 650500, China; Kunming Biomed International, Kunming, Yunnan 650500, China; State Key Laboratory of Animal Disease Control and Prevention, College of Veterinary Medicine, Lanzhou University, Lanzhou Veterinary Research Institute, Chinese Academy of Agricultural Sciences, Lanzhou, Gansu 730000, China; Key Laboratory of Veterinary Parasitology of Gansu Province, Lanzhou Veterinary Research Institute, Chinese Academy of Agricultural Sciences, Lanzhou, Gansu 730046, China; Guangxi Key Laboratory of Longevity Science and Technology, AIage Life Science Corporation Ltd., Nanning, Guangxi 530200, China; Guangxi Key Laboratory of Longevity Science and Technology, AIage Life Science Corporation Ltd., Nanning, Guangxi 530200, China; Research Center for Parasites and Vectors, College of Veterinary Medicine, Hunan Agricultural University, Changsha, Hunan 410128, China; State Key Laboratory of Animal Disease Control and Prevention, College of Veterinary Medicine, Lanzhou University, Lanzhou Veterinary Research Institute, Chinese Academy of Agricultural Sciences, Lanzhou, Gansu 730000, China; Key Laboratory of Veterinary Parasitology of Gansu Province, Lanzhou Veterinary Research Institute, Chinese Academy of Agricultural Sciences, Lanzhou, Gansu 730046, China

**Keywords:** *Blastocystis* subtypes, *Blastocystis* load, non-human primates, gut microbiome

## Abstract

*Blastocystis* is a prevalent gut eukaryote intricately associated with the gut microbiota. This genetically diverse protozoan exhibits significant intra-host subtype heterogeneity, yet the implications of this diversity for the host gut microbiome remain poorly understood. Here, we investigated the interactions between *Blastocystis* and gut microbiota in non-human primates at the level of subtypes, using a comprehensive investigation of gut microbiota for *Blastocystis* carriers of captive *Macaca fascicularis* (discovery cohort, n = 100) and *Macaca mulatta* (validation cohort, n = 26). We identified highly prevalent intra-host co-occurrence patterns of *Blastocystis SSU* rRNA-based subtypes, primarily dominated by Subtype 1 (ST1) or ST3. These patterns were associated with compositional and structural variations in the gut microbiome but were not significantly influenced by host covariates such as sex, age, or BMI. Specifically, *Ruminococcaceae*-enterotype was enriched in the patterns dominated by ST1, whereas *Limosilactobacillus*-enterotype was predominantly identified in the patterns dominated by ST3. Variance partitioning and mediation analyses revealed that the absolute abundance of *Blastocystis* was a critical determinant in elucidating this microbiota association across subtype concurrent patterns. *In vivo* experiments in a new cohort (n = 11) demonstrated that lactic acid bacteria, enriched in the *Limosilactobacillus*-enterotype, were sufficient to reduce *Blastocystis* load. We validated the strong association between gut microbiome composition and *Blastocystis* load in *M. mulatta*, confirming that specific microbial features could quantitatively predict *Blastocystis* status in both species. These findings highlight the close links of the gut microbiome with within-host subtype diversity patterns and absolute abundance of *Blastocystis*.

## Introduction

*Blastocystis* is a widely prevalent eukaryotic microorganism inhabiting the gut of both humans and animals [1]. It belongs to the heterokonts clade, which encompasses a diverse range of organisms [2]. This microorganism is globally distributed, with its prevalence significantly varying among different populations. Notably, higher prevalence rates are reported in regions with lower socio-economic development, where prevalence can reach nearly 100% [3]. The role of *Blastocystis* in host health remains unclear. Historically considered an intestinal parasite, it has been linked to multiple gastrointestinal disorders, including irritable bowel syndrome and inflammatory bowel disease [4, 5]. Some recent microbiome studies have identified *Blastocystis* as the most abundant eukaryotic commensal in the human gut [6–8]. Further research suggests that *Blastocystis* has a potentially beneficial role and is more prevalent in healthy individuals [1]. Specifically, human gut microbial communities that harbor *Blastocystis* have been associated with improved postprandial glucose responses, decreased body adiposity, favorable short-term cardiometabolic biomarkers, healthier dietary practices, and other positive health indicators [1, 9].

The discrepancies could arise from the significant genetic diversity of *Blastocystis* and its complicated links with gut microbiota. *Blastocystis* is currently categorized into at least 44 distinct subtypes (STs) with low host specificity [10, 11]. 16 subtypes are identified in humans, with ST1-ST4 being the most common [11, 12]. In non-human primates (NHPs), both zoonotic (ST1–ST5, ST7, ST8, ST10), and non-zoonotic (ST11, ST13, ST15, and ST19) have been identified [6, 13]. Notably, the co-occurrence of multiple subtypes within individuals (within-host diversity) is more common than previously thought for both animals and humans [14]. The pathogenicity of *Blastocystis* seems to be subtype-specific. For example, ST1 and ST4 can prevent DSS-induced colitis in a murine model, while ST7 exacerbates the disease [15, 16]. In addition, emerging studies have shown that *Blastocystis* colonization influences gut microbiota composition, suggesting that its influence on health and disease may be closely linked with its interactions with gut bacteria (as reviewed in [6]). These interactions are complicated by the distinct biological traits exhibited by different *Blastocystis* subtypes. Indeed, e.g. *Blastocystis* ST4 has been reported to have beneficial effects on intestinal commensal bacteria and an inhibitory role on pathogenic *Bacteroides vulgatus* [17], while ST7 exerts its pathogenic effects through disruption of the gut microbiota [18]. Thus, it has been proposed that microbial composition linked to *Blastocystis* likely depends on specific subtypes [6, 7]. Despite this, investigations into the interactions between gut microbiota and different subtypes remain largely underexplored, especially regarding their co-occurrence patterns within the host.

In this study, we explored the relationships between the gut microbiota and *Blastocystis* intra-host diversity in a discovery cohort of *Macaca fascicularis* and a validation cohort of *Macaca mulatta* at subtype resolution. Unlike the previous studies involving clinical or community-dwelling human populations, our research here strictly excluded the effects of other common intestinal parasites and took advantage of the identical parameters of captive NHPs, including genetic backgrounds, living habits, environments, and diet, to control confounding effects. Through multifaceted approaches, including experiments on a new NHP cohort, our findings provide insights into how host gut microbiota relates to both the absolute and relative abundances of *Blastocystis* in the context of within-host heterogeneity.

## Materials and methods

### Ethics declarations

All animal work was conducted according to the guidelines of the Kunming Biomed International (KBI) Animal Experiment Management and Ethics Committee (No. KBI K001123083–01, 01).

### Study cohorts and fecal sample collection

Fecal samples were collected from captive *M. fascicularis* and *M. mulatta* between 2021 and 2022 in Yuanjiang City, Yunnan Province, China. The monkeys were individually housed in separate cages (one animal per cage). The cages were cleaned before sampling to enable the collection of clean and fresh feces from each animal. Fresh samples (5–10 g/animal) were collected using a disposable sterile spoon after defecation and immediately transferred into a sterile collection tube containing DNA preservation solution (Phygene, Cat#PH1408). During sample collection, only the middle layer of feces was collected to avoid contamination. The age, sex, BMI, diet, health status, and medication history of each animal were recorded (Table S1). All samples (n = 348 for *M. fascicularis* and n = 72 for *M. mulatta*) were immediately sent to the laboratory, divided into aliquots, and stored at −80°C until use. Stool DNA was extracted using E.Z.N.A Stool DNA Kit (OMEGA, Cat#D4015–02) according to the manufacturer’s instructions.

### Inclusion and exclusion criteria

To identify *Blastocystis*, a combination of PCR-based and quantitative PCR (qPCR)–based methods was used. First, primers targeting the small subunit rRNA (*SSU* rRNA) gene (BhRDr: 5′-GAGCTTTTTAACTGCAACAACG-3′ and RD5: 5′-ATCTGGTTGATCCTGCCAGT-3′) [19] were used for screening. Second, for *Blastocystis*-negative samples, real-time qPCR was used for verification [20] (See below). Sanger sequencing on the *SSU* rRNA region was used to confirm the presence of *Blastocystis* in the positive samples in either method.

For exclusion, the common protozoans (*Cryptosporidium* spp. [21], *Giardia duodenalis* [22, 23], *Enterocytozoon bieneusi* [24], *Cyclospora* spp. [25], and Trichomonads [26]) as well as helminths (using universal primers) [27, 28] were screened using PCR methods. Nine parasite species (*Cryptosporidium hominis*, *Trichomitopsis minor*, *Pentatrichomonas hominis*, *Tetratrichomonas* sp., *Enterocytozoon bieneusi*, *Cyclospora macacae*, *Giardia duodenalis*, *Haemonchus contortus*, *Oesophagostomum muntiacum*) were found in the samples after the screening. The samples containing these pathogens were excluded. In addition, NHPs with active gastrointestinal diseases (e.g., severe diarrhea and vomiting), active chronic viral infections, or a history of antibiotics or probiotics use within the past 3 months were also excluded from further study.

### Real-time qPCR measurement

For qPCR analysis, the absolute number of *Blastocystis* cells in feces was estimated according to the previously published protocol [20]. Briefly, 2 μl of extracted DNA was mixed with 10 μl of 2× GoTaq qPCR Master Mix (Promega, Cat#A6002), 1 μl (0.5 μM) of each primer (BL18SPPF1: 5′-AGTAGTCATACGCTCGTCTCAAA-3′ and BL18SR2PP: 5′-TCTTCGTTACCCGTTACTGC-3′), and 6 μl of nuclease-free water, for a total reaction volume of 20 μl. The qPCR was performed on an Applied Biosystems (ABI) 7500 Real-Time PCR system (Thermo Fisher Scientific) with an initial denaturation step at 95°C for 2 min, followed by 45 cycles of 95°C for 15 s and 68°C for 1 min. Standard curves were established using genomic DNA with known cell numbers from an *in vitro* culture of *Blastocystis* (ST1), according to the previously reported protocol [20]. The number of *Blastocystis* cells per milligram of feces was then calculated based on the comparison of CT values in each sample against the standard curve. The *Blastocystis* load was categorized into three level groups using a framework as previously reported [29]: mild (10^0^–10^1^ ), moderate (10^1^–10^2^), and high (>10^2^). Another categorization using a different calibration range: mild (10^0^–10^1.5^), moderate (10^1.5^–10^2.5^), and high (> 10^2.5^), was also used for testing.

### Amplicon sequencing and analysis

For estimating the within-host diversity of *Blastocystis* subtypes, an amplicon sequencing strategy was employed, using a protocol as previously described [30]. In brief, 126 *Blastocystis-*positive samples were amplified using the PCR method with primers targeting a specific region of the *SSU* rRNA gene (Blast505_532F and Blast998_1017R) [31] and linked with Illumina overhang adapter sequences at the 5′ end. The final libraries were quantified using Qubit fluorometric quantitation (Thermo Fisher Scientific) and were sequenced on the NextSeq 2000 platform (Illumina), following the manufacturer’s recommendations. The paired-end reads were processed and analyzed using a pipeline that incorporates fastp (v0.19.6) [32], FLASH (v1.2.11) [33], and UPARSE (v11) [34]. The raw reads were processed using fastp to filter low-quality reads (−cut_window_size = 50, −cut_mean_quality = 20, −length_required = 50, −n_base_limit = 1). The filtered reads from each sample were merged into raw tags using FLASH (v1.2.11) (−min-overlap = 10). UPARSE was used to cluster the operational taxonomic units (OTUs) at 97% similarity and remove chimeras. The resulting *Blastocystis* OTUs were then searched by BLASTn against the NCBI nt database for subtype classification (e-value <1e^−5^). *Blastocystis* OTUs were assigned to subtypes based on their best hit in the BLASTn analysis (≥90% identity), and samples containing OTUs, which were assigned to more than one subtype, were marked as mixed-subtype infections. IQTree2 [35] was applied to construct a bootstrap maximum-likelihood tree with parameters “-m MFP -bb 1000” using the representative sequences of OTUs.

### Construction of subtype concurrent patterns of *Blastocystis*

We used a fuzzy K-means (FKM)-based method [36] to identify a distinct set of concurrent patterns of *Blastocystis* subtypes strongly supported by the relative abundance (represents the read number of each subtype relative to the total read number of *Blastocystis* in each sample) of each subtype in samples. The details are described in Supplementary Note.

### Metagenomic sequencing and analyses

DNA libraries were prepared using the NEXTFLEX Rapid DNA-Seq Kit (Bioo Scientific), following the manufacturer’s recommendations. Sequencing was performed on the NovaSeq 6000 platform (Illumina). The raw shotgun sequencing data were analyzed using the bioBakery metagenomics pipeline to generate both taxonomic and functional profiles. KneadData (v.0.10) (https://github.com/biobakery/kneaddata) was used to detect and remove reads derived from the hosts (*M. fascicularis*, genome version: GCF_012559485.2; *M. mulatta*, genome version: GCF_003339765.1). The adapter sequences at 3′ and 5′ ends of reads were trimmed, and the reads with a length less than 50 bp, an average base mass value less than 20, or having “N”-base were removed. Taxonomic annotations were performed using MetaPhlan4 [37] (v.4.0.3) using the standard reference database (mpa_vJan21_CHOCOPhlAnSGB_202103) with default parameters. The confounding covariates, including sex, age, and BMI, were controlled using MaAsLin2 for all the analyses relevant to microbiome comparisons for all the cohorts. The presence of eukaryotes was also detected using Corral [38] and EukDetect [39] with default parameters. Limited numbers of eukaryote parasites were identified in the analyses (Table S1).

### Enterotype analyses

For bacterial enterotype analysis, the gut bacterial composition was clustered at the genus level across all samples using a previously described method [40]. Briefly, bacterial taxa present in fewer than 20% of the samples were excluded from analysis to reduce noise. Clustering was conducted using partitioning around medoids (PAM) based on the Jensen-Shannon distance (JSD) between samples. The optimal number of clusters was determined using the Calinski-Harabasz index. To validate the clustering results and identify the dominant bacterial taxa in each cluster, Between-Class Analysis was performed.

### Random Forest analysis

We implemented the Random Forest (RF) regression model using the R package randomForest (v4.7–1.1) to estimate the relationship between the relative abundance of microbiota and the absolute abundance of *Blastocystis*. The original dataset of the samples from *M. fascicularis* was randomly split into a training and testing set, with a 7:3 ratio. The optimal markers to predict the absolute abundance of *Blastocystis* for the training dataset were identified using the Boruta algorithm, implemented in the R package Boruta (v8.0.0). The statistical significance (*P* value) for each marker was assessed using the R package rfPermute (v2.5.2) (https://github.com/EricArcher/rfPermute). Only markers with a *P* value ​​ < 0.05 in the permutation test were kept and then ranked by decreased %IncMSE (% Increase in Mean Squared Error). The final “bagged” RF regression model, based on the optimal set of identified microbial features, was subsequently applied to evaluate predictive efficiency in both the training and validation datasets of *M. fascicularis*. In addition, the *M. mulatta* cohort, as the independent validation cohort in this study, was also used to test the model. The performance of the regression model was assessed using the coefficient of determination (*R*^2^), which was calculated by the formula:


$$ {R}^2=1-\frac{\sum_{i=1}^n{\left({y}_i-{\hat{y}}_i\right)}^2}{\sum_{i=1}^n{\left({y}_i-{\overline{y}}_i\right)}^2} $$

${y}_i$ and ${\hat{y}}_i$ represent the actual values and predicted value of absolute abundance of *Blastocystis* in sample i. ${\underset{\_}{y}}_i$ represent the mean value of all observations (${y}_1$, ${y}_2$, ${y}_3$, ..., ${y}_i$).

### *Lactobacillus reuteri* intervention experiment

The *L. reuteri* A21041 strain was kindly provided by the Guangxi Key Laboratory of Longevity Science and Technology, Nanning. The lyophilized powder of this strain was dissolved in sterile PBS (Gibco, Cat#C20012500BT) and cultured on a MRS solid medium plate for 24 h at 37°C in anaerobic conditions to estimate the number of viable bacteria. We recruited a new group of *Blastocystis* carriers (*M. fascicularis*, n = 11) and housed them in separate cages at an ambient temperature of 22 ± 1°C and a 12/12 h light/dark cycle. The lyophilized powder of the *L. reuteri* strain was thoroughly mixed with normal saline (Servicebio, Cat#G4702) and administered via oral gavage (1×10^10^ CFU/day/animal). All *M. fascicularis* received the *L. reuteri* intervention for 21 consecutive days. Fecal samples were collected every 24 h before the intervention and qPCR was used to evaluate *Blastocystis* load in each sample.

### Statistical analysis

Statistical analyses were performed in R (v4.4.0). The “vegan” package (v2.6–8) was used for α-diversity analysis. The MaAsLin2 (v1.18) [41] package was used for controlling for confounding covariates (sex, age, and BMI) for differential abundance, group comparison, and association analyses. β-diversity was measured based on the Bray–Curtis distance (without *Blastocystis* part*)*, with statistical significance assessed via Permutational Multivariate Analysis of Variance (PERMANOVA) using the “adonis2” function in the vegan package (v2.6–8). The aPCoA package (v1.3) [42] was also used to adjust for covariates (sex, age, and BMI) in Principal Coordinates Analysis (PCoA) or PCA analysis. The distance-based redundancy analysis (dbRDA) was performed based on Bray–Curtis distance using the “dbrda” function in the vegan package (v2.6–8) (999 permutations). The function “mediate” from R package mediation was used to estimate the mediation effect. Nonparametric bootstrap procedures with 1000 simulations were performed to calculate confidence intervals and test statistical significance. Spearman’s rank correlation coefficient was computed using the “cor.test” function, and differences between groups were assessed using the Wilcoxon rank-sum test (two-sided, confidence level = 0.95). In linear regression analysis, the categorical variable is treated as ordered continuous data (Fig. S1B, Fig. S2B), and the analysis is performed in R using the lm function with the formula: (dependent variable ~ independent variable + sex + age + BMI). Benjamini-Hochberg procedure (*FDR*) was used to correct *P* values for multiple hypothesis testing.

## Results

### Cohort descriptions of NHPs

**Figure 1:**
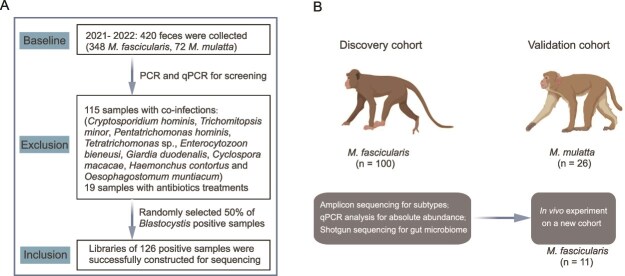
The overview of the study design. (A) Inclusion and exclusion of the samples in the study. (B) Overview of the study cohorts and methodologies employed. A discovery cohort of *Macaca fascicularis*, a validation cohort of *Macaca mulatta*, and an independent experimental cohort of *M. fascicularis* were used in this study. All the included samples were subject to a comprehensive analysis based on multifaceted techniques of PCR, qPCR, amplicon sequencing, and shotgun sequencing.

To investigate the links between *Blastocystis* and the host gut microbiome, we collected fecal samples from captive *M. fascicularis* (discovery cohort, n = 348) and *M. mulatta* (validation cohort, n = 72) in Yuanjiang City, Yunnan Province, China (Fig. 1A). All primates in both cohorts shared the same diet and living environments. The presence of *Blastocystis* in the feces was diagnosed using a combination of PCR and qPCR methods targeting the specific *SSU* rRNA gene. We observed an unexpectedly high prevalence of *Blastocystis* at 97.9% (411 out of 420 samples), illustrating its widespread occurrence in these NHPs. To control for the influence of other prevalent intestinal parasites, we employed PCR-based methods to screen for various protozoans (including *Cryptosporidium* spp., *Giardia duodenalis*, *Enterocytozoon bieneusi*, *Cyclospora* spp., and Trichomonads) and common helminths (i.e., Nematodes and tapeworms). Samples with confirmed or suspected infections, or those that met the exclusion criteria, were excluded from further analyses (Table S1). Ultimately, we included 126 *Blastocystis* carriers (n = 100 for *M. fascicularis* and n = 26 for *M. mulatta*) for further microbiome analyses (Fig. 1B).

Each sample underwent microbiome profiling via metagenomic shotgun sequencing, yielding at least 40.1 million high-quality reads. We utilized amplicon sequencing of the *SSU* rRNA to estimate within-host *Blastocystis* genetic diversity in individual carriers, which produced a minimum of 42126 reads per sample. In addition, we also determined the absolute abundance of *Blastocystis* in each sample using qPCR (Fig. 1B). Due to the identification of only nine non-carrier controls (8 *M. fascicularis* and 1 *M. mulatta*), we did not include these samples in any further analysis to avoid potential statistical bias from the unequal sample sizes. With this comprehensive dataset, we tried to gain insights into the alterations of the host gut microbiome associated with *Blastocystis*.

### Identifiable within-host subtype diversity patterns of *Blastocystis* in NHPs

**Figure 2:**
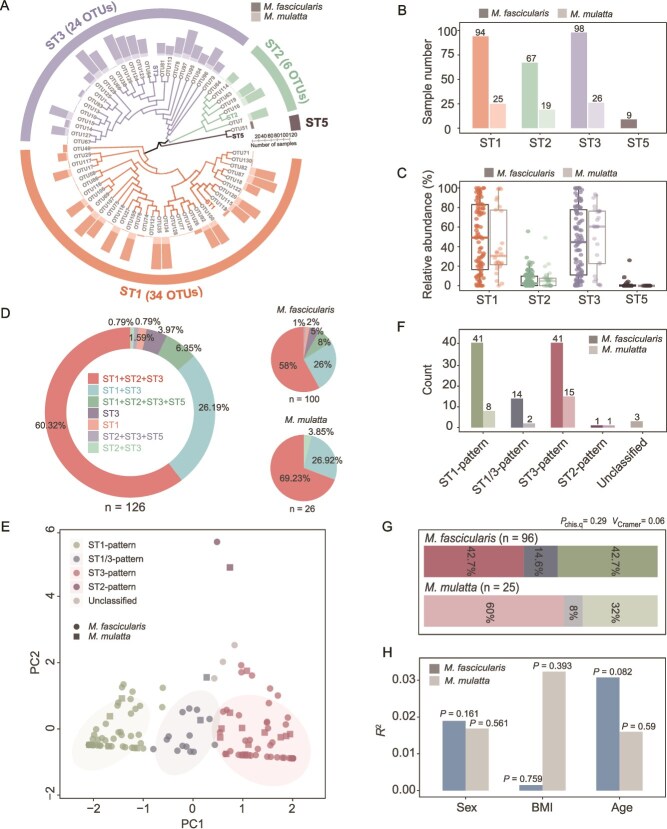
The characteristics of concurrent patterns of *Blastocystis* in NHPs. (A) Phylogenetic tree of *Blastocystis* subtype diversity at the OTU level. The OTUs with highlighted colors represent the reference sequence for each subtype. The bar plot indicates the number of samples with the presence of each subtype in amplicon sequencing. (B) The distribution of each *Blastocystis* subtype. (C) The relative abundance of each subtype in the samples. (D) The diversity of within-host subtype co-occurrence (concurrent patterns) of *Blastocystis* in NHPs. (E) The distribution of concurrent patterns. The patterns were clustered using the FKM-based method. (F) The number of samples within each pattern. (G) The prevalence of the three dominant patterns (ST1-pattern, ST1/3-pattern, and ST3-pattern). (H) The effect size and significance of each host variable (sex, BMI, and age) on PCA of concurrent patterns in PERMANOVA analysis. The *R*^2^ value represents the effect size for each variable. The *P* values were determined using the chi-squared test in panel G and using PERMANOVA analysis for panel H. The box plot represents the 25th percentile, median, and 75th percentile and whiskers stretch to 1.5 times the interquartile range from the corresponding hinge.

Currently, at least 44 subtypes of *Blastocystis* have been reported [10, 11]. Using the amplicon sequencing reads for *Blastocystis*, we identified 65 OTUs clustered at 97% identity across all samples, belonging to four subtypes: ST1, ST2, ST3, and ST5 (Fig. 2A, Fig. S3, and Table S2). The subtype assignment within each sample in the amplicon sequencing method accurately recapitulated the subtype sequence obtained by Sanger sequencing in the PCR method, with a 97.6% consistency (Table S1). We compared the prevalence and relative abundance of each subtype across the samples. We found that ST1 and ST3 were the most prevalent subtypes, appearing in 94.44% (119/126) and 98.41% (124/126) of samples, respectively (Fig. 2B). These subtypes represent the most abundant *Blastocystis* in the samples in terms of relative abundance (relative to the total number of *Blastocystis*) (Fig. 2C). Co-occurrence of these two subtypes with one another or with other subtypes within a host was notably common in this study. Specifically, the ST1 + ST2 + ST3 combination was the most prevalent, occurring in 60.32% of samples (76/126), followed by ST1 + ST3 (26.19%, 33/126), ST1 + ST2 + ST3 + ST5 (6.35%, 8/126), ST2 + ST3 (0.79%, 1/126), and ST2 + ST3 + ST5 (0.79%, 1/126) (Fig. 2D). Similar trends were observed in both *M. fascicularis* and *M. mulatta* (Fig. 2D), highlighting a high within-host subtype diversity of *Blastocystis* in NHPs.

Based on the relative abundance of the two primary subtypes in each sample, we applied the FKM-based method to robustly classify the presence of *Blastocystis* in 121 NHPs into three main patterns (hereafter referred to as “**concurrent pattern**”): ST1-pattern, ST3-pattern, and ST1/3-pattern (Fig. 2E and Fig. S4). These mixed subtype patterns are characterized by the dominance of either ST1, ST3, or a balanced presence of both, respectively. Notably, the ST1-pattern and ST3-pattern predominated over the ST1/3-pattern in terms of prevalence (Fig. 2F) within both *M. fascicularis* and *M. mulatta*, suggesting competitive interactions between subtypes within the *Blastocystis* populations (Fig. 2G). The PERMANOVA analysis indicated that differences in host parameters, including age, BMI, and sex among the NHPs could not adequately explain the clustering of observed patterns in NHPs (Fig. 2H, PERMANOVA test, *P* > 0.05). These findings support the notion that the presence of *Blastocystis* can be characterized by concurrent patterns in the carriers of both *M. fascicularis* and *M. mulatta*.

### Host gut microbiome variations characterize concurrent patterns

**Figure 3:**
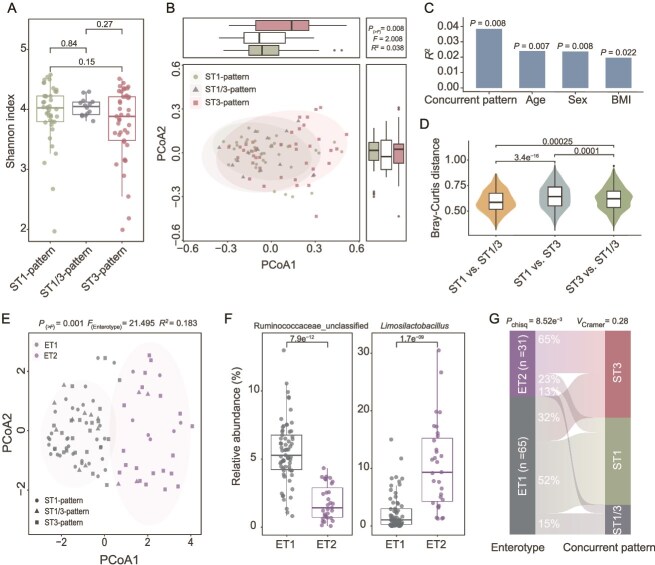
The gut microbiome across concurrent patterns in *Macaca fascicularis*. (A) The Shannon index across concurrent patterns. (B) The Principal Coordinates Analysis (PCoA) of the gut microbiota at the species level based on Bray–Curtis distances. (C) Effect sizes of concurrent patterns and sample covariates on the PCoA analysis. The *R*^2^ value for each variable was determined using the PERMANOVA analysis. (D) The Bray–Curtis distances for inter-group comparisons. (E) The gut microbiome enterotypes in concurrent patterns. Enterotypes were identified using JSD and PAM clustering at the genus level. (F) Relative abundances of Ruminococcaceae and *Limosilactobacillus* within the enterotypes. (G) Associations between enterotypes (ET1 and ET2) and concurrent patterns. The number of samples is shown in each panel. The confounding variables (sex, age, and BMI) were adjusted in the analysis for panels B and E. The *P* values were determined by the Wilcoxon rank-sum test for panels A, D, and F, by the PERMANOVA test for B, C, and E, and by the chi-squared test for panel G. The box plot represents the 25th percentile, median, and 75th percentile and whiskers stretch to 1.5 times the interquartile range from the corresponding hinge.

Next, we examined the associations between the subtype concurrent patterns of *Blastocystis* and gut microbial compositions. To streamline our analysis, we only present the findings for the discovery cohort, *M. fascicularis*, and will specify the key findings for the validation cohort, *M. mulatta* in the end. The Shannon index (Fig. 3A) indicated that the level of alpha diversity was comparable across concurrent patterns. We assessed compositional variations using PCoA based on Bray–Curtis distance at the species level and found a significant shift in the gut microbiome among *Blastocystis* subtype presence patterns (Fig. 3B, PERMANOVA test, *P* = 0.008). Furthermore, our PERMANOVA analysis revealed that the concurrent patterns accounted for more variations in gut microbiota than known host covariates such as age, BMI, and sex (Fig. 3C, PERMANOVA test, *P* = 0.008). As illustrated by the Bray–Curtis distance between samples (Fig. 3D), gut microbial compositions in ST1-pattern samples were structurally more similar to those in ST1/3-pattern samples than to those in ST3-pattern samples, indicating microbiota compositional differences across the samples with different co-occurrence of subtypes.

Furthermore, we investigated microbial variations associated with the concurrent patterns in terms of bacterial enterotype, which provides a structurally broader understanding of the relationships involved. Using JSD-PAM-based methods, we classified the gut microbiota of *M. fascicularis* into two distinct enterotypes, characterized by the dominant genera: an unclassified genus in Ruminococcaceae (ET1) and *Limosilactobacillus* (ET2) (Fig. 3E and F). PCoA analysis, based on Jensen-Shannon divergence, demonstrated a significant distinction between samples from ET1 and ET2 (PERMANOVA test, *P* = 0.001). The bacterial enterotype analysis clearly illustrated distinct associations with microbial structures across different concurrent patterns (Fig. 3G, chi-squared test, *P* = 8.52e^−3^). Specifically, ET1 was enriched in both the ST1-pattern and ST1/3-pattern, whereas ET2 was predominantly identified in the ST3-pattern (Fig. 3G). Additionally, the differential analysis conducted using MaAsLin2, while controlling for confounding covariates such as age, sex, and BMI, identified numerous species (n = 24) with significant differences in abundance between concurrent patterns in *M. fascicularis* (Fig. S5). Most of these species were not assigned to a known genus in the database. Notably, the ST1 pattern exhibited a significant shift, with 18 microbiota species differing from those in the ST3 pattern (Fig. S5). This data indicates a substantial variation in gut microbial composition among the different concurrent patterns. Collectively, these findings suggest that the subtype concurrent patterns of *Blastocystis* are correlated with compositional and structural variations in the gut microbiome.

### *Blastocystis* load mediates the associations between concurrent patterns and gut microbiota

**Figure 4:**
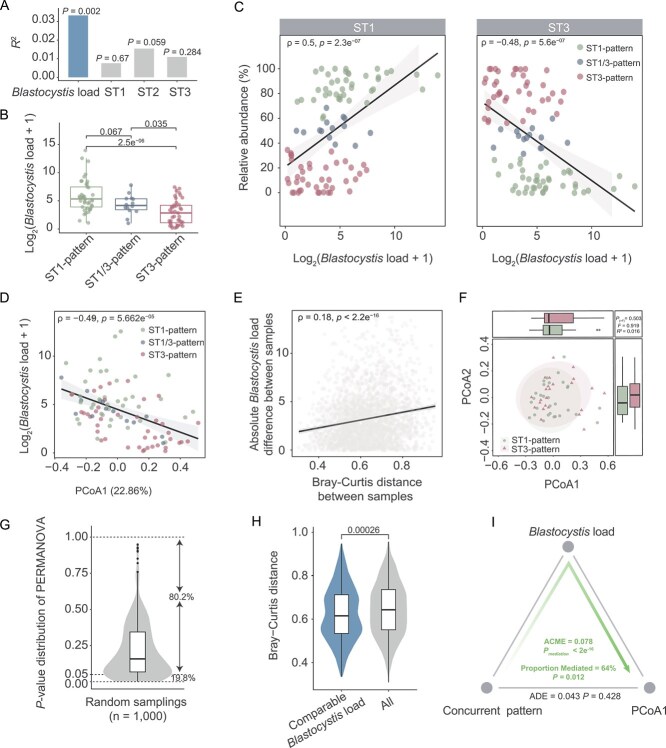
*Blastocystis* load explains the associations between concurrent patterns and the gut microbiota in *Macaca fascicularis*. (A) Effect sizes of the absolute abundance of *Blastocystis* and relative abundance of each subtype related to concurrent patterns on the PCoA analysis of the gut microbiota (Fig. 3B) in the PERMANOVA analysis. (B) The absolute abundance of *Blastocystis* in each concurrent pattern. Log2-transformed values are shown for the Y-axis. (C) Spearman correlation between the relative abundance of ST1 (or ST3) and the total *Blastocystis* load in the sample. (D) Spearman correlation between *Blastocystis* load and the first principal coordinate (PCoA1) in PCoA analysis (Fig. 3B). (E) Spearman correlation between Bray–Curtis distance and *Blastocystis* load. (F) Bray–Curtis distance-based PCoA analysis for samples of ST1-pattern and ST3-pattern with the comparable *Blastocystis* load between them. (G) Distribution of *P* values from the PERMANOVA analysis by bootstrap sampling (n = 1000). (H) Bray–Curtis distances between ST1-pattern and ST3-pattern for samples with comparable absolute abundance. (I) The mediation effect analysis shows the relationship between concurrent patterns, the PCoA1 of the gut microbiome and *Blastocystis* load in ST1- and ST3-pattern. The confounding variables (sex, age, and BMI) were adjusted in the analysis for panels D and F. The *P* values were determined by the PERMANOVA test for panels A and F, by the Wilcoxon rank-sum test for panels B and H, by MaAsLin2 for panel D, and by mediation analysis for panel I. Spearman’s rank correlation coefficient test was used for panels C and E. The box plot represents the 25th percentile, median, and 75th percentile. Whiskers stretch to 1.5 times the interquartile range from the corresponding hinge.

To gain insights into the association between concurrent patterns and the gut microbiome in *M. fascicularis*, we examined factors related to the co-occurrence of subtypes, specifically the abundance of each subtype. Our analysis revealed that the total *Blastocystis* load in each sample exhibited the strongest explanatory power for variations in gut microbiota (Fig. 4A, PERMANOVA test, *P* = 0.002). This finding suggests that the absolute abundance of *Blastocystis* in each sample represents a critical feature in differentiating concurrent patterns. Indeed, qPCR analysis indicated that samples exhibiting the ST1 or ST1/ST3 patterns contained significantly higher amounts of *Blastocystis* compared to those with the ST3-pattern (Fig. 4B, Wilcoxon rank-sum test, *P* < 0.05). Notably, we found a significant positive correlation between the relative abundance of the ST1 subtype and *Blastocystis* load across samples, while the relative abundance of ST3 demonstrated a negative correlation with *Blastocystis* levels (Fig. 4C, Spearman’s rank correlation coefficient test, *P* = 2.3e^−07^ and *P* = 5.6e^−07^, respectively). This suggests that the dominant subtype within each concurrent pattern influences the overall quantity of *Blastocystis* present.

Thus, our data suggest that the absolute abundance of *Blastocystis* is a key factor explaining the associations between gut microbial variations and concurrent patterns. Supporting this idea, we observed a significant correlation between the first principal coordinate (PCoA1) in the PCoA analysis (Fig. 3B) and the measured *Blastocystis* count in feces (Fig. 4D, MaAsLin2, *P* = 5.662e^−05^). Additionally, the Bray–Curtis dissimilarities in gut microbiomes between samples were significantly correlated with variations in absolute abundance of *Blastocystis* (Fig. 4E, Spearman’s rank correlation coefficient test, *P* < 2.2e^−16^). To further investigate whether *Blastocystis* load dominates the associations between gut microbiome and concurrent patterns, we randomly selected ST1-pattern samples (n = 15) with a *Blastocystis* load comparable to that of ST3-pattern samples (n = 15) and compared their microbiome structures using PERMANOVA through a bootstrap resampling method (n = 1000). PCoA analysis indicated that microbial structural differences diminished between the two patterns (Fig. 4F and G, PERMANOVA, *P* > 0.05, Bootstrap = 80.2%), accompanied by a significant reduction in Bray–Curtis distance (Fig. 4H).

Using dbRDA, we further tested the statistical significance of the variance explained by concurrent patterns and total *Blastocystis* load. As expected, *Blastocystis* load had a significant independent effect on gut microbial beta diversity (*P* = 0.002), whereas concurrent patterns showed no significant independent contribution after controlling for *Blastocystis* load (*P* = 0.473) (Table S3). All these data support the idea that the link between concurrent patterns and the gut microbiome might be mediated by the *Blastocystis* load. We thus tested this hypothesis using a causal mediation analysis and confirmed a mediator effect from the absolute abundance of this eukaryote on the variations of the gut bacterial part (Fig. 4I, *P* < 2e^−16^). In addition, the direct effect of *Blastocystis* concurrent patterns on gut microbial beta-diversity was not observed (*P* = 0.428).

To minimize bias from the quantitative method used to determine absolute abundance, we reanalyzed our data by categorizing the samples into three groups based on *Blastocystis* load: mild (10^0^–10^1^ cells/mg feces), moderate (10^1^-10^2^ cells/mg feces), and high (>10^2^ cells/mg feces). We found no significant differences in gut microbiota among the concurrent patterns within the same load group in the PCoA. The correlation between PCoA1 of the gut microbiome and the measured *Blastocystis* count in feces diminished for samples within the same *Blastocystis* load level (Fig. S1). In contrast, we observed a significant association between PCoA1 and the groups with different load levels. Additionally, the Bray–Curtis distances between samples within the same load level were significantly lower than those between different load groups, further indicating an association between gut microbiome and *Blastocystis* load. Furthermore, we tested another calibration range: 10^0^–10^1.5^ (mild), 10^1.5^–10^2.5^ (moderate), and > 10^2.5^ (high), and obtained similar results (Fig. S2).

Collectively, these findings support the idea that *Blastocystis* load is a critical determinant in elucidating the associations between concurrent patterns and gut microbiota.

### Lactic acid bacteria reduce the absolute abundance of *Blastocystis* in carriers

**Figure 5:**
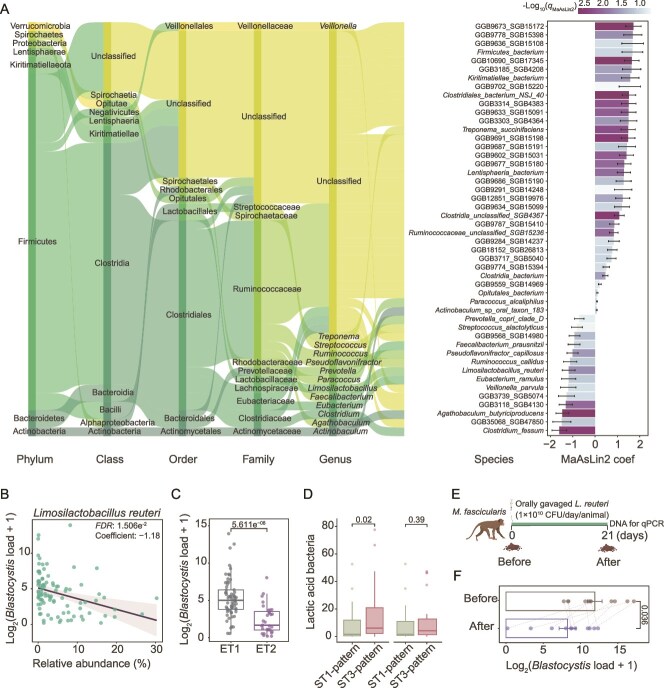
Impact of lactic acid bacteria on *Blastocystis* load in *Macaca fascicularis*. (A) The associations of microbial taxa at the species level with *Blastocystis* load in MaAsLin2 analysis. The top taxa ranked by q-value (q < 0.1) are shown at different levels. (B) Correlation between the relative abundance of *Limosilactobacillus reuteri* and *Blastocystis* load in MaAsLin2 analysis. (C) Comparison of *Blastocystis* load between samples of enterotypes (ET1 and ET2). (D) Relative abundance of lactic acid bacteria (*Lactobacillus* and *Limosilactobacillus*) in ST1- and ST3-pattern for the samples with comparable *Blastocystis* load. (E) Experimental design for an independent cohort of *M. fascicularis*. Oral administration of *Lactobacillus reuteri* was performed daily. (F) Changes of *Blastocystis* load before and post the experiment. qPCR was used to examine the *Blastocystis* abundance in feces. The confounding variables (sex, age, and BMI) were adjusted in the analysis for panels A, B, and C using MaAsLin2. The *P* values or *FDR* were calculated using MaAsLin2 for panels A, B, and C, the Wilcoxon rank-sum test for panel D, and the Wilcoxon signed-rank test for panel F. The box plot represents the 25th percentile, median, and 75th percentile and whiskers stretch to 1.5 times the interquartile range from the corresponding hinge. The data of mean ± SD is shown for panels A and F.

Our above results suggest a close relationship between host gut microbiota and the abundance of *Blastocystis*; we thus investigated the microbial factors influencing *Blastocystis* load in carriers. We assessed the association between *Blastocystis* load and gut microbial compositions in *M. fascicularis* while controlling for other host covariates (BMI, sex, and age) using MaAsLin2. At the species level, we identified 92 bacteria that demonstrated a strong co-occurrence with *Blastocystis* (Table S4 and Fig. 5A). Notably, species from the lactic acid bacteria group (*Limosilactobacillus* and *Lactobacillus*) ranked among the most enriched taxa that were negatively associated with *Blastocystis* load (Table S4). For instance, *Limosilactobacillus reuteri* emerged as the predominant taxon (Fig. 5B). This aligns with our observation that ET2, enriched in *Limosilactobacillus*, exhibited a significantly lower *Blastocystis* load compared to enterotype ET1, which is enriched in Ruminococcaceae (Fig. 5C, MaAsLin2, *P* = 5.611e^−08^). Moreover, we found the level of lactic acid bacteria in the ST1-pattern was significantly lower than that in the ST3-pattern, whereas this trend disappeared if samples with comparable *Blastocystis* load were randomly selected for comparison (Fig. 5D, Wilcoxon rank-sum test, *P* = 0.02 and *P* > 0.05, respectively).

These findings support the hypothesis that lactic acid bacteria could impact *Blastocystis* levels in primates. Indeed, a previous study has indicated that lactic acid bacteria may inhibit the proliferation of *Blastocystis in vitro* [43]. To test this hypothesis in NHPs *in vivo*, we recruited a new cohort of *Blastocystis* carriers of *M. fascicularis* (n = 11) and administered an *L. reuteri* strain (1 × 10^10^ CFU/day/animal) orally. We measured the absolute level of *Blastocystis* in their feces using qPCR (Fig. 5E). After 21 days of *L. reuteri* gavage, we observed a significant reduction in *Blastocystis* number compared to the pre-treatment levels (Fig. 5f, Wilcoxon signed-rank test, *P* < 0.05).

### The relationship between *Blastocystis* load and the gut microbiota is a common and predictive feature in NHPs

**Figure 6:**
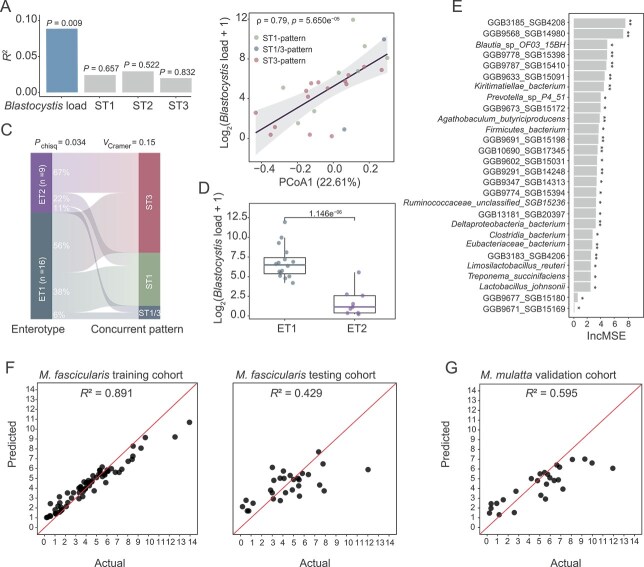
The gut microbiota composition can predict *Blastocystis* load in NHPs. (A) Effect sizes of each variable on the PCoA analysis of the gut microbiota for *Macaca mulatta.* PERMANOVA analysis was used to determine each variable’s effect size and significance. (B) Spearman correlation between *Blastocystis* load and PCoA1 of the microbial analysis in *M. mulatta*. (C) Associations between enterotypes (ET1 and ET2) and concurrent patterns (ST1/3, ST1, and ST3) in *M. mulatta*. (D) *Blastocystis* load in the samples with ET1 and ET2 in *M. mulatta*. (E) The identified markers (n = 28) in RF analysis for the discovery cohort dataset (n = 71). These markers are ranked based on their percentage increase in mean squared error (%IncMSE). (F) The performance of the RF model in predicting the training or testing cohort of *Macaca fascicularis*. (G) The performance of the RF model in predicting the independent validation cohort of *M. mulatta*. The confounding variables (sex, age, and BMI) were adjusted in the analysis for panels B and D using MaAsLin2. The *P* values were determined using PERMANOVA analysis for panel A, MaAsLin2 for panels B and D, and the chi-squared test for panel C. ^*^P value < 0.05; ^**^P value < 0.01.

Our findings indicate that the gut microbial compositions are closely linked to the abundance of *Blastocystis* in *M. fascicularis*. This observation raised questions about whether this association is a common feature that could serve as an indicator of *Blastocystis* levels in NHPs. To explore this hypothesis, we first replicated our analyses in the cohort of *M. mulatta*. Using the same analytical methods and controlling for the confounding factors (sex, age, and BMI), we identified comparable trends to those seen in *M. fascicularis*, confirming that *Blastocystis* load significantly correlates with microbial variations across different concurrent patterns (Fig. 6A–C). This conclusion is supported by extensive analyses of compositional and structural configurations, particularly regarding diversity and bacterial enterotypes (Fig. 6A–C and Fig. S6). Consistently, we found that the *Blastocystis* load was significantly lower in gut microbiota configurations characterized by a high abundance of *Limosilactobacillus* (Fig. 6D, MaAsLin2, *P* = 5.650e^−05^). Furthermore, we developed a machine-learning classifier utilizing the RF method, partitioning the samples from *M. fascicularis* into discovery and testing subsets. The microbial biomarkers identified (Fig. 6E) were able to effectively predict *Blastocystis* load in both testing and validation datasets of *M. fascicularis*, achieving a high coefficient of determination (*R^2^*) (Fig. 6F). Of note, this RF model was also able to efficiently predict the abundance of *Blastocystis* for the independent validation cohort of *M. mulatta* (Fig. 6G). These results suggest that certain microbial features associated with *Blastocystis* load are potentially consistent across the two NHP species. Collectively, this data supports the hypothesis that the gut microbiome plays a predictive role regarding *Blastocystis* load in its carriers.

## Discussions

*Blastocystis* is a widespread protist found in both animals and humans globally. The notably high prevalence of *Blastocystis* in NHPs, specifically the 97.9% presence rate recorded in our study, highlights its ecological significance within primate gut microbiomes. Thus, understanding the relationships between *Blastocystis* and the other parts within the host gut microbiome is of high importance. Given the substantial inter-subtype genetic variation, characterizing these subtypes is essential for understanding the connections between microbiota composition and *Blastocystis*.

To the best of our knowledge, this study is the first cohort investigation focusing on the impact of within-host mixed-subtype of *Blastocystis* on the gut microbiota. Our findings highlight the significant prevalence of mixed-subtype existence within a single host in NHPs. Although often underexplored, previous studies indicate that the presence of mixed subtypes within a specimen is more common than previously recognized [14, 30, 44, 45]. For example, co-occurrence involving multiple *Blastocystis* subtypes was identified in 62.5% of positive samples from birds [46] and 22% of human cases [14]. In our study, employing an amplicon sequencing method [30], we discovered that the co-occurrence of multiple subtypes accounts for over 90% of the total carriers involved. Additionally, our results revealed a high intra-subtype variability and a substantial number of sequence variants even at the OTU level. Given our findings, along with previous research, consistently demonstrate a strong correlation between subtypes and microbial variation, it is crucial to consider intra-subtype and even intra-isolate genetic heterogeneity in *Blastocystis*-related microbiome studies.

Our findings underscore the critical role of absolute abundance in examining the interplay between *Blastocystis* and gut microbiota. Numerous studies have reported subtype-dependent associations with gut microbiota, with different subtypes exhibiting varying effects in both human and murine models [6, 7]. For instance, the presence of ST4 was correlated with elevated levels of *Sporolactobacillus* and *Candidatus carsonella* in Swedish travelers, while ST3 did not demonstrate such significant relationships [47]. Additionally, an inverse correlation between ST3 and ST4 and *Akkermansia* abundance was observed in fecal samples from a distinct cohort study [7]. These findings highlight the complex nature of *Blastocystis*, as different subtypes possess divergent biological characteristics, including growth rates, colonization niches, and host preferences [18, 48]. However, the underlying mechanisms remain largely unclear. In this study, we dissected that the absolute abundance of *Blastocystis* is a key determinant for understanding concurrent pattern associations with gut microbiota. This may reflect the biological variations, particularly in growth rates or colonization dynamics among subtypes. This is in line with findings in a cohort study of human populations, which indicated a higher load in ST1 carriers compared to ST3 carriers [7], as well as an *in vitro* study that demonstrated a higher growth rate for ST1 than for ST3 when cultured in DMEM medium [49].

Our findings provide insights into the relationship between the absolute abundance of *Blastocystis* and the gut microbiota. Notably, our results suggest that this relationship cannot be solely attributed to the amount-dependent effects of *Blastocystis*. Instead, our data indicate that the load of *Blastocystis* may be influenced, at least in part, by the host’s microbial composition, particularly the presence of lactic acid bacteria. Our *in vivo* experiment demonstrated a strong inhibitory effect of lactic acid bacteria on *Blastocystis* levels in NHPs. This aligns with previous research, which found that *in vitro* co-incubation with either *Lactobacillus rhamnosus, L. lactis*, or *Enterococcus faecium* inhibited the growth of *Blastocystis* [43]. These findings strongly suggest that a higher abundance of these beneficial bacteria may enhance a host’s resistance to *Blastocystis* colonization, although the precise underlying mechanisms require further investigation. Furthermore, it is reasonably speculated that the presence of *Blastocystis* in primates can lead to alterations in microbial composition and diversity, as reported in various studies on different models. Nonetheless, this research underscores the potential of using *L. reuteri* as a prophylactic approach to regulate *Blastocystis* colonization.

Our study has several limitations. First, we did not include negative controls in the gut microbiome analysis. The prevalence of *Blastocystis* in our cohorts was relatively high, with only nine negative samples identified out of hundreds screened. Due to this small sample size, we avoided directly comparing positive and negative groups to prevent statistical bias. Second, in our *in vivo* experiment with *L. reuteri*, the sample size was limited, and no placebo control group was included for comparison. Further investigation will be necessary in future studies to address these issues.

In summary, this study underscores the ecological significance of *Blastocystis* in primate guts and emphasizes the need to consider subtype diversity and absolute abundance in microbiome studies. The inhibitory role of lactic acid bacteria opens avenues for probiotic interventions in NHPs. Future research should explore mechanistic pathways and validate findings in human cohorts and wild populations.

## Supplementary Material

Figure_S1-6_ycaf170(1)

Supplemenatry_tables_ycaf170(1)

Supplemenatry_Note_ycaf170

## Data Availability

Data are available in the main text or the supplementary materials. Sequences have been deposited to NCBI with accession number: PRJNA1222911. Original R scripts, metadata, and OTU tables are available on GitHub at (https://github.com/Axolotl233/Blastocystis_microbes).

## References

[1] Piperni E, Nguyen LH, Manghi P. et al. Intestinal *Blastocystis* is linked to healthier diets and more favorable cardiometabolic outcomes in 56,989 individuals from 32 countries. *Cell* 2024;187:4554–70.e18. 10.1016/j.cell.2024.06.018 38981480

[2] Derelle R, López-García P, Timpano H. et al. A phylogenomic framework to study the diversity and evolution of Stramenopiles (=heterokonts). *Mol Biol Evol* 2016;33:2890–8. 10.1093/molbev/msw168 27512113 PMC5482393

[3] El Safadi D, Gaayeb L, Meloni D. et al. Children of Senegal River basin show the highest prevalence of *Blastocystis* sp. ever observed worldwide. *BMC Infect Dis* 2014;14:164. 10.1186/1471-2334-14-164 24666632 PMC3987649

[4] Poirier P, Wawrzyniak I, Vivarès CP. et al. New insights into *Blastocystis* spp.: a potential link with irritable bowel syndrome. *PLoS Pathog* 2012;8:e1002545. 10.1371/journal.ppat.1002545 22438803 PMC3305450

[5] Cifre S, Gozalbo M, Ortiz V. et al. *Blastocystis* subtypes and their association with irritable bowel syndrome. *Med Hypotheses* 2018;116:4–9. 10.1016/j.mehy.2018.04.006 29857906

[6] Deng L, Wojciech L, Gascoigne NRJ. et al. New insights into the interactions between *Blastocystis*, the gut microbiota, and host immunity. *PLoS Pathog* 2021;17:e1009253. 10.1371/journal.ppat.1009253 33630979 PMC7906322

[7] Tito RY, Chaffron S, Caenepeel C. et al. Population-level analysis of *Blastocystis* subtype prevalence and variation in the human gut microbiota. *Gut* 2019;68:1180–9. 10.1136/gutjnl-2018-316106 30171064 PMC6582744

[8] Andersen LO, Bonde I, Nielsen HB. et al. A retrospective metagenomics approach to studying *Blastocystis*. *FEMS Microbiol Ecol* 2015;91:91. 10.1093/femsec/fiv072 26130823

[9] Asnicar F, Berry SE, Valdes AM. et al. Microbiome connections with host metabolism and habitual diet from 1,098 deeply phenotyped individuals. *Nat Med* 2021;27:321–32. 10.1038/s41591-020-01183-8 33432175 PMC8353542

[10] Stensvold CR, Clark CG. Pre-empting pandora's box: *Blastocystis* subtypes revisited. *Trends Parasitol* 2020;36:229–32. 10.1016/j.pt.2019.12.009 32001133

[11] Šejnohová A, Koutenská M, Jirků M. et al. A cross-sectional survey of *Blastocystis* sp. and *Dientamoeba fragilis* in non-human primates and their caregivers in Czech zoos. *One Health* 2024;19:100862. 10.1016/j.onehlt.2024.100862 39157650 PMC11327577

[12] Popruk S, Adao DEV, Rivera WL. Epidemiology and subtype distribution of *Blastocystis* in humans: a review. *Infect Genet Evol* 2021;95:105085. 10.1016/j.meegid.2021.105085 34530156

[13] Köster PC, Martínez-Nevado E, González A. et al. Intestinal protists in captive non-human primates and their handlers in six European zoological gardens. Molecular evidence of zoonotic transmission. *Front Vet Sci* 2021;8:819887. 10.3389/fvets.2021.819887 35059456 PMC8763706

[14] Scanlan PD, Stensvold CR, Cotter PD. Development and application of a *Blastocystis* subtype-specific PCR assay reveals that mixed-subtype infections are common in a healthy human population. *Appl Environ Microbiol* 2015;81:4071–6. 10.1128/aem.00520-15 25841010 PMC4524157

[15] Deng L, Wojciech L, Png CW. et al. Colonization with two different *Blastocystis* subtypes in DSS-induced colitis mice is associated with strikingly different microbiome and pathological features. *Theranostics* 2023;13:1165–79. 10.7150/thno.81583 36793854 PMC9925320

[16] Deng L, Wojciech L, Png CW. et al. Colonization with ubiquitous protist *Blastocystis* ST1 ameliorates DSS-induced colitis and promotes beneficial microbiota and immune outcomes. *NPJ Biofilms Microbiomes* 2023;9:22. 10.1038/s41522-023-00389-1 37185924 PMC10130167

[17] Deng L, Tan KSW. Interactions between *Blastocystis* subtype ST4 and gut microbiota *in vitro*. *Parasit Vectors* 2022;15:80. 10.1186/s13071-022-05194-x 35260166 PMC8902775

[18] Yason JA, Liang YR, Png CW. et al. Interactions between a pathogenic *Blastocystis* subtype and gut microbiota: *in vitro* and *in vivo* studies. *Microbiome* 2019;7:30. 10.1186/s40168-019-0644-3 30853028 PMC6410515

[19] Scicluna SM, Tawari B, Clark CG. DNA barcoding of *blastocystis*. *Protist* 2006;157:77–85. 10.1016/j.protis.2005.12.001 16431158

[20] Poirier P, Wawrzyniak I, Albert A. et al. Development and evaluation of a real-time PCR assay for detection and quantification of *blastocystis* parasites in human stool samples: prospective study of patients with hematological malignancies. *J Clin Microbiol* 2011;49:975–83. 10.1128/jcm.01392-10 21177897 PMC3067686

[21] Koehler AV, Korhonen PK, Hall RS. et al. Use of a bioinformatic-assisted primer design strategy to establish a new nested PCR-based method for *cryptosporidium*. *Parasit Vectors* 2017;10:509. 10.1186/s13071-017-2462-4 29061171 PMC5654123

[22] Lalle M, Pozio E, Capelli G. et al. Genetic heterogeneity at the beta-giardin locus among human and animal isolates of *Giardia duodenalis* and identification of potentially zoonotic subgenotypes. *Int J Parasitol* 2005;35:207–13. 10.1016/j.ijpara.2004.10.022 15710441

[23] Cacciò SM, Beck R, Lalle M. et al. Multilocus genotyping of *Giardia duodenalis* reveals striking differences between assemblages A and B. *Int J Parasitol* 2008;38:1523–31. 10.1016/j.ijpara.2008.04.008 18571176

[24] Buckholt MA, Lee JH, Tzipori S. Prevalence of *Enterocytozoon bieneusi* in swine: an 18-month survey at a slaughterhouse in Massachusetts. *Appl Environ Microbiol* 2002;68:2595–9. 10.1128/aem.68.5.2595-2599.2002 11976142 PMC127518

[25] Li W, Kiulia NM, Mwenda JM. et al. *Cyclospora papionis*, *Cryptosporidium hominis*, and human-pathogenic *Enterocytozoon bieneusi* in captive baboons in Kenya. *J Clin Microbiol* 2011;49:4326–9. 10.1128/jcm.05051-11 21956988 PMC3232936

[26] Kleina P, Bettim-Bandinelli J, Bonatto SL. et al. Molecular phylogeny of Trichomonadidae family inferred from ITS-1, 5.8S rRNA and ITS-2 sequences. *Int J Parasitol* 2004;34:963–70. 10.1016/j.ijpara.2004.04.004 15217735

[27] Cannon MV, Bogale H, Rutt L. et al. A high-throughput sequencing assay to comprehensively detect and characterize unicellular eukaryotes and helminths from biological and environmental samples. *Microbiome* 2018;6:195. 10.1186/s40168-018-0581-6 30373673 PMC6206884

[28] Bowles J, Blair D, McManus DP. Genetic variants within the genus *Echinococcus* identified by mitochondrial DNA sequencing. *Mol Biochem Parasitol* 1992;54:165–73. 10.1016/0166-6851(92)90109-w 1435857

[29] Šloufová M, Lhotská Z, Jirků M. et al. Comparison of molecular diagnostic approaches for the detection and differentiation of the intestinal protist *Blastocystis* sp. in humans. *Parasite* 2022;29:30. 10.1051/parasite/2022029 35638752 PMC9153396

[30] Maloney JG, Molokin A, Santin M. Next generation amplicon sequencing improves detection of *Blastocystis* mixed subtype infections. *Infect Genet Evol* 2019;73:119–25. 10.1016/j.meegid.2019.04.013 31026606

[31] Santín M, Gómez-Muñoz MT, Solano-Aguilar G. et al. Development of a new PCR protocol to detect and subtype *Blastocystis* spp. from humans and animals. *Parasitol Res* 2011;109:205–12. 10.1007/s00436-010-2244-9 21210149

[32] Chen S, Zhou Y, Chen Y. et al. fastp: an ultra-fast all-in-one FASTQ preprocessor. *Bioinformatics* 2018;34:i884–90. 10.1093/bioinformatics/bty560 30423086 PMC6129281

[33] Magoč T, Salzberg SL. FLASH: fast length adjustment of short reads to improve genome assemblies. *Bioinformatics* 2011;27:2957–63. 10.1093/bioinformatics/btr507 21903629 PMC3198573

[34] Edgar RC . UPARSE: highly accurate OTU sequences from microbial amplicon reads. *Nat Methods* 2013;10:996–8. 10.1038/nmeth.2604 23955772

[35] Minh BQ, Schmidt HA, Chernomor O. et al. Corrigendum to: IQ-TREE 2: new models and efficient methods for phylogenetic inference in the genomic era. *Mol Biol Evol* 2020;37:2461. 10.1093/molbev/msaa131 32556291 PMC7403609

[36] Magdalena L . Fuzzy rule-based systems. In: Kacprzyk J, Pedrycz W (eds.), Springer Handbook of Computational Intelligence. 2015. Springer Berlin Heidelberg, Berlin, Heidelberg, pp. 203–18. doi:10.1007/978-3-662-43505-2_13

[37] Blanco-Míguez A, Beghini F, Cumbo F. et al. Extending and improving metagenomic taxonomic profiling with uncharacterized species using MetaPhlAn 4. *Nat Biotechnol* 2023;41:1633–44. 10.1038/s41587-023-01688-w 36823356 PMC10635831

[38] Bazant W, Blevins AS, Crouch K. et al. Improved eukaryotic detection compatible with large-scale automated analysis of metagenomes. *Microbiome* 2023;11:72. 10.1186/s40168-023-01505-1 37032329 PMC10084625

[39] Lind AL, Pollard KS. Accurate and sensitive detection of microbial eukaryotes from whole metagenome shotgun sequencing. *Microbiome* 2021;9:58. 10.1186/s40168-021-01015-y 33658077 PMC7931531

[40] Mu WJ, Ma PP, Wang YG. et al. Taeniasis impacts human gut microbiome composition and function. *ISME J* 2024;18:18. 10.1093/ismejo/wrae213 PMC11536184 39441994

[41] Mallick H, Rahnavard A, McIver LJ. et al. Multivariable association discovery in population-scale meta-omics studies. *PLoS Comput Biol* 2021;17:e1009442. 10.1371/journal.pcbi.1009442 34784344 PMC8714082

[42] Shi Y, Zhang L, Do KA. et al. aPCoA: covariate adjusted principal coordinates analysis. *Bioinformatics* 2020;36:4099–101. 10.1093/bioinformatics/btaa276 32339223 PMC7332564

[43] Lepczyńska M, Dzika E. The influence of probiotic bacteria and human gut microorganisms causing opportunistic infections on *Blastocystis* ST3. *Gut Pathog* 2019;11:6. 10.1186/s13099-019-0287-8 30815037 PMC6376780

[44] Scanlan PD, Stensvold CR. *Blastocystis*: getting to grips with our guileful guest. *Trends Parasitol* 2013;29:523–9. 10.1016/j.pt.2013.08.006 24080063

[45] Rojas-Velázquez L, Maloney JG, Molokin A. et al. Use of next-generation amplicon sequencing to study *Blastocystis* genetic diversity in a rural human population from Mexico. *Parasit Vectors* 2019;12:566. 10.1186/s13071-019-3814-z 31775832 PMC6882168

[46] Maloney JG, Molokin A, da Cunha MJR. et al. *Blastocystis* subtype distribution in domestic and captive wild bird species from Brazil using next generation amplicon sequencing. *Parasite Epidemiol Control* 2020;9:e00138. 10.1016/j.parepi.2020.e00138 32021915 PMC6995250

[47] Forsell J, Bengtsson-Palme J, Angelin M. et al. The relation between *Blastocystis* and the intestinal microbiota in Swedish travellers. *BMC Microbiol* 2017;17:231. 10.1186/s12866-017-1139-7 29228901 PMC5725903

[48] Tan KS . New insights on classification, identification, and clinical relevance of *Blastocystis* spp. *Clin Microbiol Rev* 2008;21:639–65. 10.1128/cmr.00022-08 18854485 PMC2570156

[49] Karamati SA, Mirjalali H, Niyyati M. et al. Comprehensive study of phenotypic and growth rate features of *Blastocystis* subtypes 1-3 and 6 in symptomatic and asymptomatic subjects. *Iran J Parasitol* 2019;14:204–13. 31543908 PMC6737371

