# Supplementary material for: Blastocystis load mediates the gut microbiome associations with within-host diversity of Blastocystis in non-human primates
Source: ISME Commun. 2025 Sep 26;5(1):ycaf170. doi: 10.1093/ismeco/ycaf170 (PMC12560790; doi:10.1093/ismeco/ycaf170)
Supplement: Figure_S1-6_ycaf170(1) [file figure_s1-6_ycaf170(1).pdf]

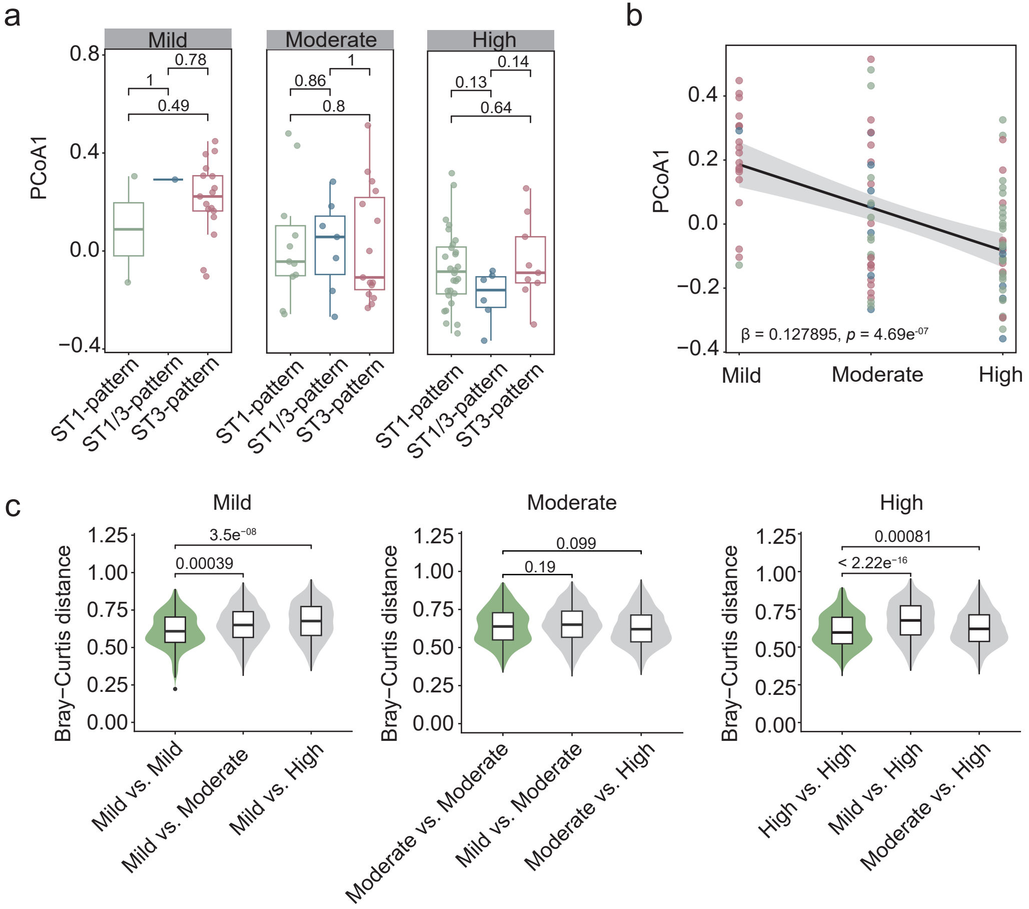

Figure S1. Microbial community structure and composition associated with different *Blastocystis* load levels (mild: 10^0-10^1, moderate: 10^1-10^2, and high: >10^2). (a) The first principal coordinate (PCoA1) in different concurrent patterns. **(b)** Linear regression (LM) of the PCoA1 and different *Blastocystis* load levels in the sample. **(c)** Inter-group differences of Bray-Curtis distances between samples with different *Blastocystis* load levels. The *P* values were determined by the Wilcoxon rank-sum test for panels a and c, and by the linear regression for panel b. The box plot represents the 25th percentile, median, and 75th percentile and whiskers stretch to 1.5 times the interquartile range from the corresponding hinge.

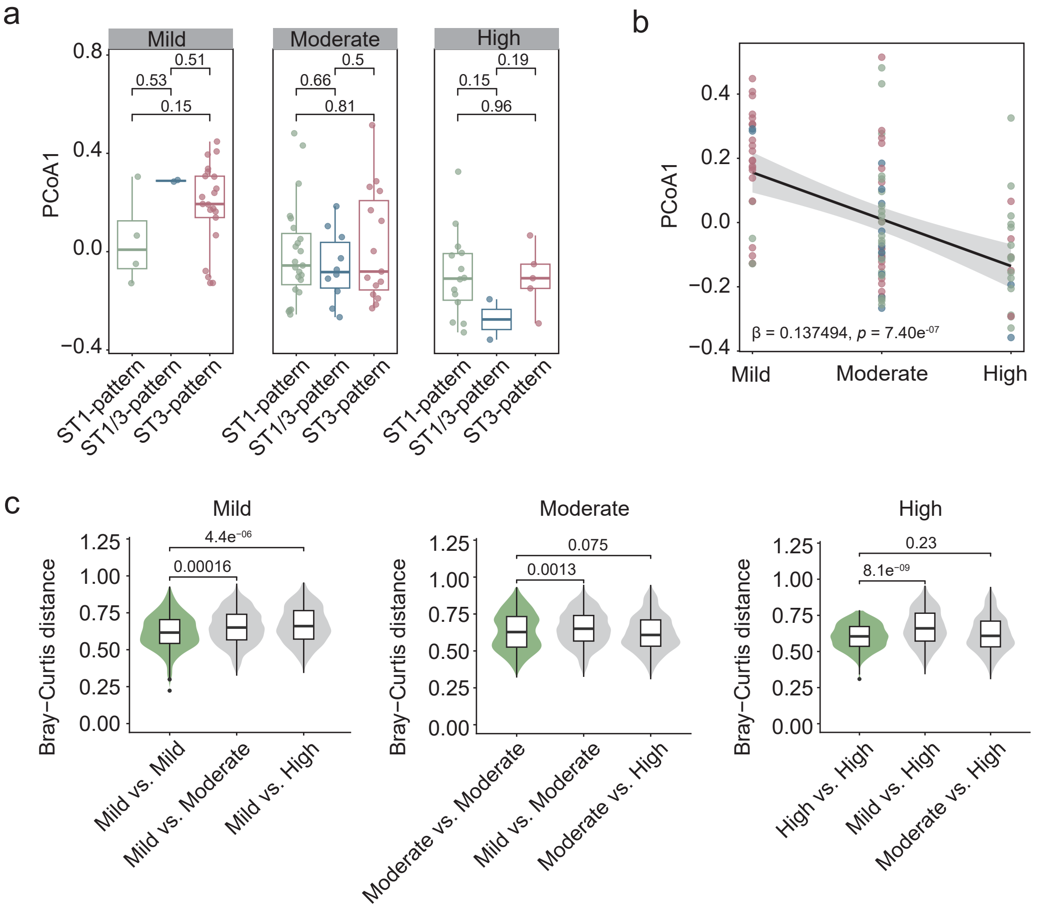

Figure S2. Microbial community structure and composition associated with different *Blastocystis* load levels (mild: 10^{0}-$10^{1.5}$, moderate: $10^{1.5}$-$10^{2.5}$, and high: >$10^{2.5}$). (a) The first principal coordinate (PCoA1) in different concurrent patterns. **(b)** Linear regression (LM) of the PCoA1 and different *Blastocystis* load levels in the sample. **(c)** Inter-group differences of Bray-Curtis distances between samples with different *Blastocystis* load levels. The *P* values were determined by the Wilcoxon rank-sum test for panels a and c, and by the linear regression for panel b. The box plot represents the 25th percentile, median, and 75th percentile and whiskers stretch to 1.5 times the interquartile range from the corresponding hinge.

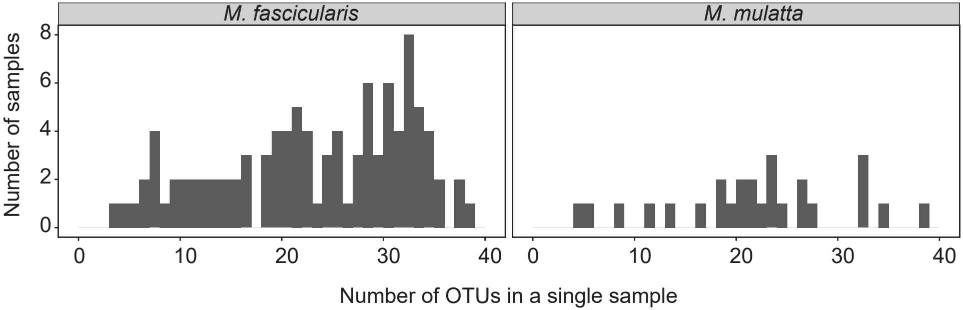

Figure S3. The number of OTUs in individual samples of NHPs.

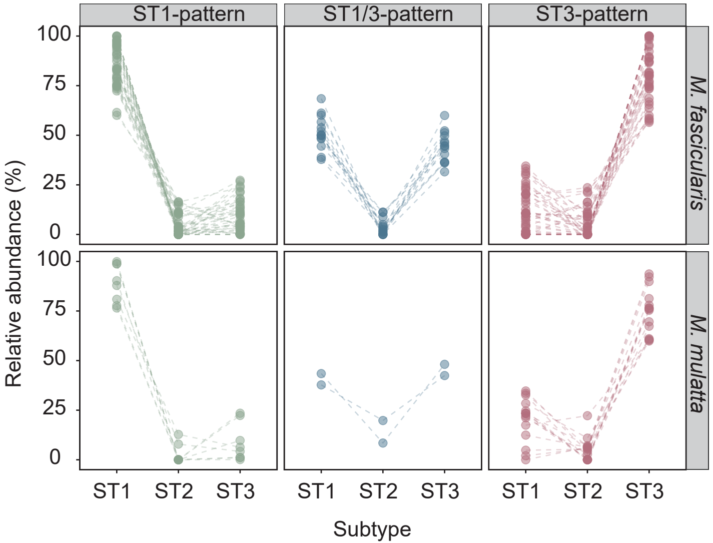

Figure S4. The relative abundance of each *Blastocystis* subtype within each concurrent pattern in NHPs.

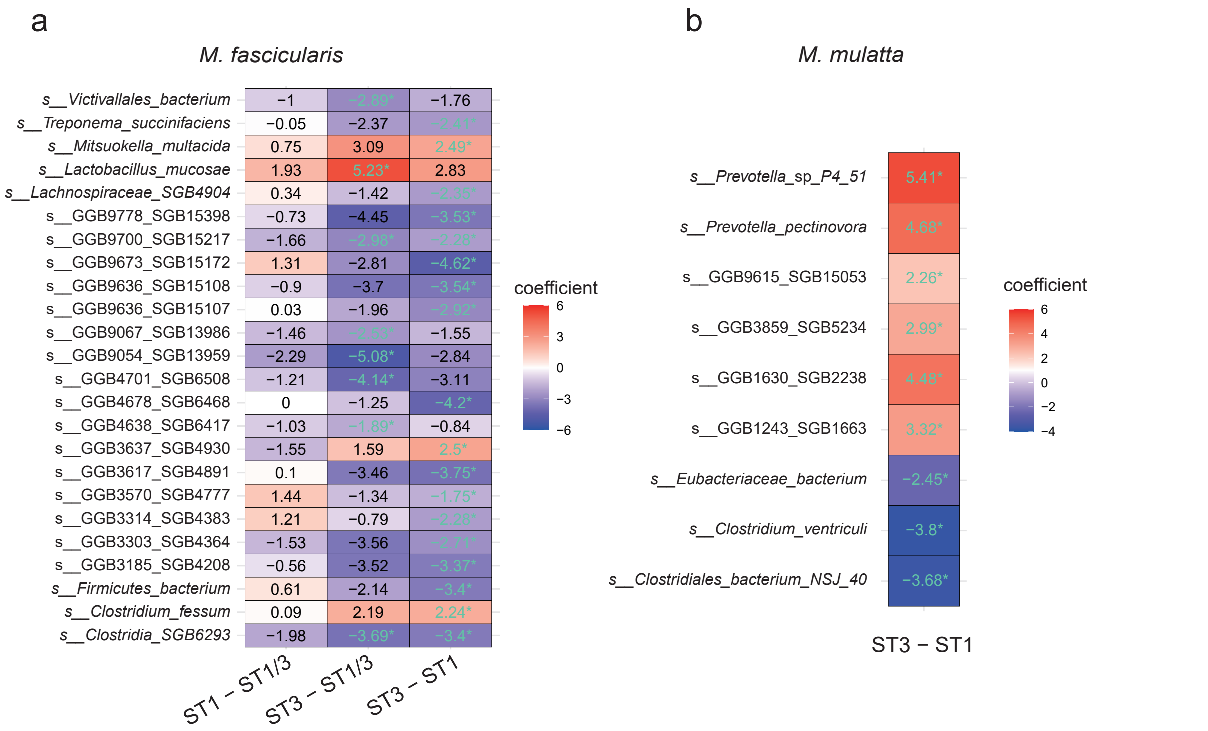

Figure S5. Differential abundance analysis at the species-level between concurrent patterns in *M. fascicularis* and *M. mulatta*. The heatmap indicated species with significant differential abundances in comparisons for *M. fascicularis* **(a)** and *M. mulatta* **(b)**. Color and label of heatmap represent coefficient values. * indicates the significance test passed the default cutoff of *FDR* in the MaAsLin2 analysis.

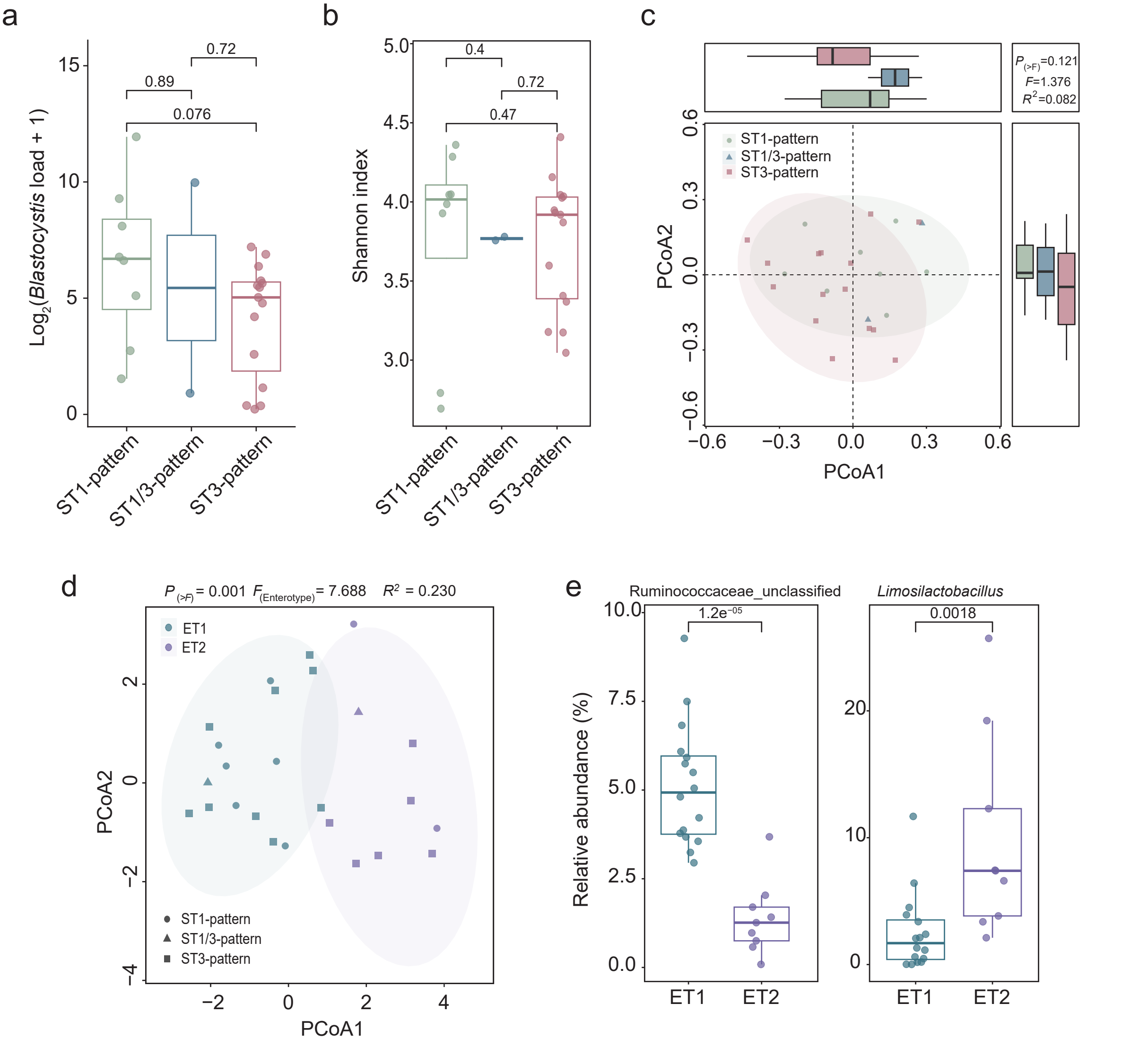

Figure S6. The associations between the gut microbiome and *Blastocystis* load across different patterns in *M. mulatta*. (a) Absolute abundance of *Blastocystis* in each concurrent pattern. Log2-transformed values are displayed on the Y-axis. **(b)** The Shannon index across subtype concurrent patterns. **(c)** The Principal Coordinates Analysis (PCoA) of the gut microbiota at the species level, based on Bray-Curtis distances. **(d)** The gut microbiome enterotypes in concurrent patterns. Enterotypes were identified using Jensen-Shannon distance (JSD) and partitioning around medoid (PAM) clustering at the genus level. **(e)** The relative abundances of Ruminococcaceae and *Limosilactobacillus* within the identified enterotypes. The analysis for panels c and d was adjusted for confounding variables, including sex, age, and BMI. The *P* values were determined using PERMANOVA analysis for panels c and d, and the Wilcoxon rank-sum test for panels, a, b, and e. The box plot represents the 25th percentile, median, and 75th percentile and whiskers stretch to 1.5 times the interquartile range from the corresponding hinge.
