# Supplementary material for: Blastocystis load mediates the gut microbiome associations with within-host diversity of Blastocystis in non-human primates
Source: ISME Commun. 2025 Sep 26;5(1):ycaf170. doi: 10.1093/ismeco/ycaf170 (PMC12560790; doi:10.1093/ismeco/ycaf170)
Supplement: Supplemenatry_Note_ycaf170 [file supplemenatry_note_ycaf170.docx]

**Construction of subtype concurrent patterns of *Blastocystis***

We used a fuzzy K-means (FKM)-based method to identify a distinct set of concurrent patterns of *Blastocystis* subtypes strongly supported by the relative abundance (represents the read number of each subtype relative to the total read number of *Blastocystis* in each sample) of each subtype in samples. This method employs the FKM algorithm to initially cluster the samples into preliminary groups, followed by further filtering. The dissimilarity distance matrix used in the FKM clustering was computed using the formula:

$$d_{i, j}=\frac{1-\rho(i, j)}{2}$$

Where $d_{i, j}$ and $\rho(i, j)$ represents the distance and Pearson correlation between samples i and j, respectively. The ‘fanny’ function from R package ‘cluster’ was used to perform initial fuzzy clustering. Samples were filtered based on a membership threshold (0.85). Similar clusters were merged from the initial clustering to achieve more robust results through hierarchical clustering: (1) For each cluster, the median relative abundance of each subtype was estimated based on all assigned samples. This resulted in multiple sets of subtype medians (termed cluster-specific profiles), with the number of sets corresponding to the selected K. (2) A dissimilarity matrix was constructed based on the cluster-specific profiles, using the formula: 1 - Pearson correlation coefficient. (3) Hierarchical clustering was performed based on dissimilarity matrix, and clusters with linkage heights below 0.1 (i.e., Pearson correlation > 0.9) were merged into a single cluster. (4) We recalculated the cluster-specific profiles for each *Blastocystis* subtype within the samples of each merged cluster. This generated the final clusters and their corresponding cluster-specific profiles. Each sample was reassigned to the closest final cluster based on its Pearson correlation between the relative abundance of subtypes and the cluster-specific profiles. The Pearson correlation threshold in this step is 0.85. We tested the number of clusters (k) ranging from 1 to 6 to create the initial FKM cluster, and the optimal k = 5 was determined as the one that obtained the largest number of samples classified. The FKM-based clustering using only *M. fascicularis* resembled the same co-occurrence patterns (2 different in 100 samples) and almost the sample assignments to each pattern as those obtained from the clustering of the full NHP datasets.
